# Fecal microbiota transplantation and replenishment of short-chain fatty acids protect against chronic cerebral hypoperfusion-induced colonic dysfunction by regulating gut microbiota, differentiation of Th17 cells, and mitochondrial energy metabolism

**DOI:** 10.1186/s12974-022-02675-9

**Published:** 2022-12-26

**Authors:** Shao-Hua Su, Yi-Fang Wu, Qi Lin, Lin Zhang, Da-Peng Wang, Jian Hai

**Affiliations:** 1 Department of Neurosurgery, School of Medicine, Tongji Hospital, Tongji University, 389 Xincun Road, Shanghai, 200065 China; 2 Department of Pharmacy, School of Medicine, Institutes of Medical Sciences, Shanghai Jiao Tong University, Shanghai, 200025 China; 3 Department of Neurosurgery, School of Medicine, Shanghai Sixth People’s Hospital, Shanghai Jiao Tong University, Shanghai, 200003 China

**Keywords:** Chronic cerebral hypoperfusion, Gut microbiota, short-chain fatty acids, Mitochondrial energy metabolism, Treg/Th17 balance

## Abstract

**Background:**

Little is known about the association between gut microbiota and intestinal injury under a state of chronic cerebral hypoperfusion (CCH). Here, the effects of gut microbiota and short-chain fatty acids (SCFAs), as important metabolic products, on intestinal function and potential mechanisms after CCH were investigated.

**Methods:**

Rats were subjected to bilateral common carotid artery occlusion (BCCAo) to induce CCH. The gut microbiota and metabolites of SCFAs were assessed by 16S rRNA sequencing and targeted metabolomics, respectively. Transcriptomic analysis of colon tissues was also conducted. Subsequently, potential molecular pathways and differentially expressed genes were verified by western blot, immunoprecipitation, and immunofluorescence analyses. Furthermore, the integrity of the colonic barrier was evaluated by hematoxylin and eosin and mucin 2 staining and expression levels of tight junction proteins. Besides, colonic inflammation was further assessed by flow cytometry and expression levels of inflammatory cytokines. In addition, colonic mitochondrial dysfunction was analyzed via membrane potential, reactive oxygen species, electron transport chain (ETC) activities, adenosine triphosphate content, and mitochondrial ultrastructure.

**Results:**

CCH modified gut microbial composition and microbial metabolism of SCFAs, which may be associated with inhibition of mitochondrial ETC activities and oxidative phosphorylation, leading to dysregulation of mitochondrial energy metabolism. Furthermore, CCH induced differentiation of pathogenic Th17 cells, promoted the formation of complexes of interferon regulatory factor 4 and signal transducer and activator of transcription 3 (STAT3), and increased the phosphorylation of STAT3. This was associated with an impairment of colonic barrier function and chronic colonic inflammation. In contrast, FMT and SCFA replenishment ameliorated CCH-induced gut microbial dysbiosis by increasing the intestinal content of *Ruminococcus_sp_N15_MGS_57* and modulating microbial metabolism of SCFAs by increasing acetic acid contents associated with an improvment of the balance between Tregs and Th17 cells, mitochondrial ETC activities, and oxidative phosphorylation to prevent colonic inflammation and dysregulation of mitochondrial energy metabolism.

**Conclusion:**

These findings indicate that FMT and SCFA replenishment present a promising therapeutic strategy against colonic dysfunction under a state of chronic cerebral ischemia.

## Introduction

Chronic cerebral hypoperfusion (CCH), which refers to a chronic state of reduced cerebral blood flow, is associated with the pathology of various cerebrovascular diseases and cognitive impairment via cerebral ischemia-induced apoptosis, inflammation, and excessive autophagy [1–5]. Hence, protection against CCH presents a potentially effective therapeutic strategy for chronic cerebral ischemia (CCI). Possible targets for CCH intervention include the endocannabinoid system, cholinergic neuronal system, adiponectin, and leptin receptor [3, 6–8]. However, such treatments are only aimed at neuronal injury induced by cerebral ischemia. CCI not only leads to intracranial injury, but also gastrointestinal complications, such as constipation, fecal incontinence, and gastrointestinal bleeding [9]. Although gastrointestinal symptoms associated with CCI have recently attracted considerable attention, further investigations are needed to elucidate the underlying mechanisms.

The gut microbiota consists of a diverse community of bacterial species, existing symbiotically with the human host. The health and stability of the gut microbiota is vital to maintain homeostatic balance. Increasing evidence supports the functional effects of the microbiota on bidirectional communication along the microbiota-gut-brain axis [10]. Studies have highlighted the influence of the gut microbiota on the gut-brain axis and potential roles in diseases of the central nervous system, such as multiple sclerosis, Parkinson’s disease, autism, Alzheimer's disease, depression, and schizophrenia [11–14]. However, the roles of the gut microbiota in these diseases remain controversial. Although recent studies have confirmed that ischemic stroke induces changes to the composition of the gut microbiota [15, 16], little is known about the association between modified gut microbiota and intestinal injury due to CCI and the mechanisms underlying the regulation of intestinal function by the gut microbiota modified by CCH have not been characterized. Hence, a better understanding of these interactions might provide new therapeutic targets for the treatment of CCH-induced gut dysfunction.

Short-chain fatty acids (SCFAs) are important products of gut metabolism, mainly consisting of acetate, propionate, and butyrate [17], derived from fermentation of dietary and nutritional components by the gut microbiota. Both oral replenishment and fecal microbiota transplantation (FMT) can increase the content of SCFAs in the gut to protect against gut dysfunction [18]. However, to the best of our knowledge, the possible effects and mechanisms of FMT and SCFAs on CCH-induced intestinal damage have not yet been elucidated. How gut microbiota and SCFAs directly or indirectly exert effects on CCH-induced intestinal injury has yet to be uncovered. Thus, clarification of the effects of the gut microbiota and SCFAs on intestinal function may have important implications for the development of microbial-based therapeutic strategies for treatment of intestinal disorders after CCI.

Therefore, the aim of the present study was to investigate the effects of the gut microbiota and SCFAs on intestinal function and to explore potential mechanisms after CCH in order to provide new insights into the treatment of CCI-induced gut dysfunction.

## Materials and methods

### Animals and experimental protocols

Male Sprague–Dawley rats (age, 5 weeks; body weight, 180 ± 10 g) were purchased from the Shanghai Laboratory Animal Resource Center (Shanghai, China) and Shanghai SIPPR-Bk Lab Animal Co., Ltd. (Shanghai, China). Prior to experimentation, the rats were housed in a climate-controlled facility at a constant temperature of 23 ± 1 °C and 60% humidity under a 12-h day/night cycle (lights on 08:30 am–08:30 pm) with ad libitum access to water and standard rat chow. After 1 week of acclimatization, the rats were randomly assigned to one of four groups [5 rats per group for targeted metabolomics of SCFAs; 3 rats per group for RNA sequencing; 5 rats per group for histopathological analysis or immunofluorescence staining (3 rats per group for dihydroethidium (DHE) staining); 4 rats per group for western blots or immunoprecipitation (IP) (3 rats per group for IP); 5 rats per group for enzyme-linked immunosorbent assay (ELISA) or biochemical tests; 3 rats per group for flow cytometry]: (1) sham-operated group; (2) bilateral common carotid artery occlusion (BCCAo) group; (3) BCCAo + FMT group; or (4) BCCAo + SCFAs group.

**Fig. 1:**
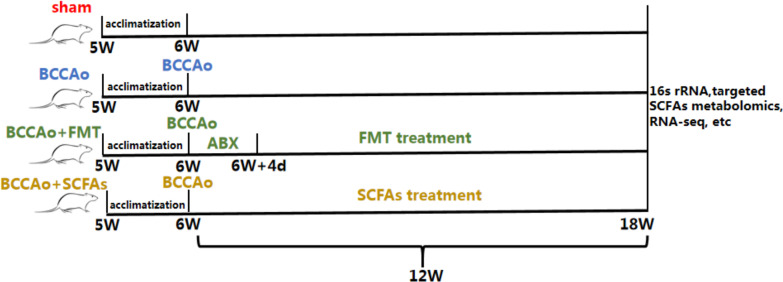
Timeline for the experimental procedure. Rats were randomly divided into four groups including sham, BCCAo, BCCAo + FMT and BCCAo + SCFAs. Rats received different interventions in these four groups for 12 weeks, then were sacrificed for subsequent experiments. *ABX* antibiotic treatment

Rats in all four groups were sacrificed after 12 weeks. Fresh feces and colon tissues were immediately collected for experiments or stored at − 80 °C for later use (Fig. 1). All experiments involving animals were approved by the Committee for Animal Experimentation of Tongji Hospital (Shanghai, China) and conducted in compliance with the Guide for the Care and Use of Laboratory Animals.

### BCCAo procedure

BCCAo at 8–12 weeks is sufficient to sustain CCH in rats and most closely resembles reduced CBF in humans [19]. In this study, 12 weeks was chosen as the maximum chronic hypoperfusion phase of CCH. As described in our previous study [3], each rat was anesthetized by intraperitoneal injection of pentobarbital-sodium (50 mg/kg). A midline incision was made to expose the bilateral common carotid arteries, which were tightly double ligated with 5–0 silk sutures. Rats in the sham-operated group underwent the same procedure, but without bilateral ligation of the common carotid arteries.

### Antibiotic treatment and FMT

As described in a previous study [20], rats in the BCCAo + FMT group received antibiotic treatment (vancomycin, 100 mg/kg; neomycin sulfate, 200 mg/kg; metronidazole, 200 mg/kg; and ampicillin, 200 mg/kg) intragastrically daily for 4 days to deplete the gut microbiota. For FMT experiments: fresh feces from stool samples were collected from each rat in the sham-operated group on the morning and mixed together. Then, the mixed feces from sham-operated group were re-suspended in sterile physiological saline solution to 100 mg/mL and immediately used for FMT. Homogenates were then passed through a nylon filter (pore size, 20 μm) to remove large particulate and fibrous matter. The fecal solution was collected and the recipient rats received 2 mL daily for 12 days by gastric gavage [21]. To avoid that the intestinal microbiota after intervention would gradually become similar to the initial state both structurally and functionally over time [22], gastric gavage was repeated once every 3 days for 12 weeks.

### SCFA treatment

As previously described [23], rats in the BCCAo + SCFAs group received a cocktail of SCFAs (sodium acetate, 67.5 mM; sodium propionate, 25.9 mM; and sodium butyrate, 40 mM; Sigma-Aldrich Corporation, St. Louis, MO, USA) in sterile phosphate-buffered saline (PBS) at 0.1 mL/10 g of body weight via gastric perfusion for 12 weeks [24].

### Gastric gavage

For gastric perfusion, a tube was carefully inserted into the stomach from the mouth (distance, 5–7 cm), while maintaining the head and neck in a straight line, and either drugs or liquified fecal samples were slowly injected through a syringe. After removing the tube, the posture of the rat was maintained for 30 s. If the rat struggled or experienced troubled breathing, the procedure was discontinued and the tube was immediately extracted. When the rat recovered to calm and breathed evenly, the procedure of gastric gavage was conducted again.

### 16S rRNA gene sequencing

The 16S rRNA genes of snap-frozen fecal samples were sequenced by BioNovoGene Co., Ltd. (Suzhou, China). Briefly, total DNA was extracted from fecal samples (250 mg, wet weight) using the QIAamp DNA Stool Mini Kit (Qiagen GmbH, Hilden, Germany) in accordance with the manufacturer’s instructions. DNA integrity and size were verified by 1% agarose gel electrophoresis and DNA concentrations were determined using a NanoDrop spectrophotometer (NanoDrop Technologies, LLC, Wilmington, DE, USA). For each DNA sample, 16S rRNA was amplified with the primers 341F (5’-CCT AYG GGR BGC ASC AG-3’)/806R (5’-GGA CTA CNN GGG TAT CTA AT-3’), which directionally target the V3 and V4 hypervariable regions. Each 20 μL PCR reaction volume contained 4 μL of 5 × FastPfu Buffer, 2 μL of 2.5 mM dNTPs, 0.8 μL of each primer (5 μM), 0.4 μL of FastPfu DNA Polymerase, and 10 ng of template DNA. The PCR reaction included an initial denaturation step at 95 °C for 3 min, followed by 27 cycles of denaturation at 95 °C for 30 s, annealing at 55 °C for 30 s, and elongation at 72 °C for 45 s, and a final extension step at 72 °C for 10 min. The reactions were performed on a GeneAmp™ PCR System 9700 (Applied Biosystems, Carlsbad, CA, USA). All PCR products were purified using an AxyPrep™ DNA Gel Extraction Kit (Axygen Scientific, Inc., Union City, CA, USA) and quantified using a QuantiFluor™-ST fluorometer (Promega Corporation, Madison, WI, USA). Normalized equimolar concentrations of the purified amplicons were pooled and sequenced with a NovaSeq PE250 sequencing instrument (Illumina, Inc., San Diego, CA, USA) in accordance with the manufacturer’s specifications.

High-throughput sequencing analysis of the bacterial 16S rRNA genes was processed using Quantitative Insights into Microbial Ecology software (version 1.9.1) [25]. The chimeric sequences were filtered using the USEARCH sequence analysis tool (Uparse software v6.0.307). Sequences with similarity thresholds > 97% were allocated to one operational taxonomic unit using the clustering algorithm Cluster Database at High Identity with Tolerance (v4.6.1). The alpha diversity value was calculated to determine the species diversity of the samples, which was evaluated with the observed_species and Shannon diversity indices. Beta diversity was used to assess differences in species diversity among the samples and characterized by non-metric multi-dimensional scaling (NMDS). At the genus level, the Tukey and Wilcoxon rank-sum tests were used to compare bacterial abundance and diversity. Analysis of similarities was performed as a non-parametric test to identify differences in community structures and species among groups [26]. Heat maps were constructed based on the nonparametric Wilcoxon test using MetaStat (*p* < 0.05). The linear discriminant analysis effect size (LEfSe) method was applied to evaluate the differentially abundant taxa.

16S rRNA gene sequencing data were deposited into the BioProject database (https://www.ncbi.nlm.nih.gov/bioproject) under the accession number PRJNA869931.

### Targeted metabolomics of SCFAs

Targeted metabolomics analysis of SCFAs of snap-frozen fecal samples and colon tissues was performed by BioNovoGene Co., Ltd. Briefly, 100 mg of feces and 300 mg of colon tissues were dispersed in acidified water spiked with stable isotope-labeled SCFA standards and extracted with diethyl ether. The ether layer was immediately analyzed by gas chromatography-mass spectrometry (GC-MS) with a TRACE 1300 gas chromatograph (Thermo Fisher Scientific, Waltham, MA, USA). The SCFA standards used in this study were mixtures of acetate, propionate, butyrate, isobutyrate, valerate, isovalerate, and caproate. Quantitation was performed by calibration to internal standards. The protein contents of homogenized tissues were normalized. Then, samples (1 μL) were injected at a split ratio of 10:1. Helium was used as the carrier gas at a constant flow rate of 1.0 mL/min. The injection, transfer line, and ion source temperatures were set at 250 °C, 250 °C, and 230 °C, respectively. The initial temperature program was set at 2 min of isothermal heating at 90 °C and then increased to 120 °C at a rate of 10 °C/min, then to 150 °C at 5 °C/min and finally to 250 °C at 25 °C/min, which was maintained for 2 min. Data were acquired in full-scan mode (electron impact ionization, 70 eV) with an m/z range of 35–780.

The GC-MS data obtained in the.raw format from the platform were converted to the.mzXML format using the msConvert tool ((ProteoWizard). Then, the data sets were normalized, transformed, imputed, and scaled after outlier removal using the R package (v3.3.2). The levels of seven SCFA metabolites were quantified and compared with the non-parametric Mann–Whitney U test. Orthogonal partial least squares discriminant analysis (OPLS-DA) was applied to identify differences among groups.

### RNA sequencing and data deposition

RNA extracted from snap-frozen colon tissues were sequenced by BioNovoGene Co., Ltd. Briefly, samples with an RNA integrity number > 8 were submitted for library prep and next-generation sequencing. cDNA libraries were prepared using a NEBNext® Ultra™ II RNA Library Prep Kit for Illumina® (New England Biolabs, Ipswich, MA, USA). Purified cDNA libraries were sequenced using a NovaSeq PE250 sequencing instrument with > 6G raw reads (150 bp, pair-ended) per sample. Differentially expressed genes (DEGs) were identified by RNA sequencing (RNA-seq) based on the criteria *p* < 0.05 and |log_2_fold change|> 0.5 for pathway analysis with the Kyoto Encyclopedia of Genes and Genomes (KEGG), Gene Ontology (GO) analysis, and gene set enrichment analysis (GSEA).

RNA-Seq data were deposited into the BioProject database (https://www.ncbi.nlm.nih.gov/bioproject) under the accession number PRJNA781099.

### Isolation of mitochondria

The mitochondrial fraction was isolated using the Qproteome® Mitochondria Isolation Kit (Qiagen GmbH). Briefly, colon tissues (~ 20 mg) were cut into pieces and homogenized in 2 mL of lysis buffer with protease inhibitor solution. The supernatant was centrifuged at 1000 × *g* and 4 °C for 10 min. Then, the resulting pellet was resuspended and disrupted in 1.5 mL of ice-cold disruption buffer. Following centrifugation at 1000 × *g* and 4 °C for 10 min, the supernatant was collected and centrifuged at 6000 × *g* for 10 min. The pellet (containing mitochondria) was resuspended in 750 μL of mitochondrial purification buffer and added on the top of a mitochondrial purification buffer layer. After centrifugation at 14,000 × *g* for 15 min, the pellet or band containing mitochondria that formed in the lower part of the tube was transferred to a new tube. The suspension was washed three times with 1.5 mL of mitochondrial storage buffer by centrifugation at 8000 × *g* for 10 min. The high-purified mitochondria were resuspended in mitochondrial storage buffer and used to determine the membrane potential. The remaining mitochondrial and cytosolic fractions were stored at − 80 °C for later use.

### Mitochondrial membrane potential

The membrane potential of isolated mitochondria was measured using a JC-1 staining kit (Beyotime Institute of Biotechnology, Haimen, China). Briefly, 10 μL of fresh mitochondria were incubated in 100 μL of JC-1 staining reagent for 10 min. Images were captured using an inverted microscope (IX71; Olympus Corporation, Tokyo, Japan) at wavelengths of 488 and 594 nm. The ratio of red/green intensity was observed to assess the membrane potential of isolated mitochondria. Healthy mitochondria showed a high intensity of red fluorescence at 594 nm and a low intensity of green fluorescence at 488 nm, while impaired mitochondria demonstrated opposite results.

### Colonic adenosine triphosphate (ATP) content and electron transport chain (ETC) complex I–V activities

Colon tissues were homogenized in ice-cold HEPES buffer (3 mM, pH 7.2) containing sucrose (0.25 M), egtazic acid (0.5 mM), and protease inhibitor cocktail (1:40; Roche Life Science, Penzberg, Germany). The protein concentration of the tissue homogenate was measured with a Pierce™ BCA Protein Assay Kit ( Pierce Biotechnology, Waltham, MA, USA). Then, the fractions were quantified using ATP and ETC Complex I–V assay kits (Beijing Solarbio Science & Technology Co., Ltd., Beijing, China) in accordance with the manufacturer’s instructions. Briefly, equal amounts of fresh proteins were loaded into all wells and absorbance was measured using a spectrophotometer as follows: ε340 nm for nicotinamide adenine dinucleotide (NADH) dehydrogenase (complex I) and ATP content, ε550 nm for mitochondrial complex III (cytochrome c reductase) or complex IV (cytochrome c oxidase), ε605 nm for complex II (succinate-coenzyme Q reductase), and ε660 nm for complex V (F0F1-ATPase/ATP synthase). For convenience, the ATP content is expressed as µmol/mL, while the other results are expressed as µmol/mg of protein.

### Histopathological analysis and immunofluorescence staining

Paraffin-embedded sections were deparaffinized and washed three times with PBS for 5 min for histopathological analysis and immunofluorescence staining. For histopathological analysis, the slices were stained with a hematoxylin and eosin (HE) staining kit (Baso Diagnostics, Inc., Wuhan, China) in accordance with the manufacturer’s instructions and viewed under an inverted microscope (Olympus IX71) to identify morphological changes to the intestinal mucosa. For immunofluorescence staining, the sections were immersed in ethylenediaminetetraacetic acid (EDTA)-Tris solution (pH 9.0) for 30 min at 98 °C for antigen retrieval, rinsed three times with PBS for 5 min, and then incubated with 10% non-immune goat serum for 30 min at room temperature to block non-specific labeling before overnight incubation with an antibody against mucin 2 (MUC2) (1:50; Santa Cruz Biotechnology, Inc., Dallas, TX, USA) in a humidified chamber at 4 °C [27]. After washing with PBS, the sections were incubated with tetraethylrhodamine isothiocyanate-conjugated secondary antibodies (1:200; Santa Cruz Biotechnology, Inc.) for 1 h at 37 °C. MUC2-positive spots in colon tissues were imaged with a laser scanning confocal microscope (LSM 700; Carl Zeiss AG, Oberkochen, Germany).

### DHE staining

Reactive oxygen species (ROS) were detected by staining with DHE. Briefly, the sections were incubated in 10 mM DHE (Beyotime Institute of Biotechnology) at room temperature for 30 min in the dark and then viewed under an inverted microscope (Olympus IX71).

### ELISA

The amount of acetyl coenzyme A (Ac-CoA) in colon tissues was quantified with a commercial ELISA kit (Nanjing Jiancheng Bioengineering Institute, Nanjing, China) in accordance with the manufacturer’s instructions. Briefly, 50 mg tissues were homogenized and centrifuged at 4000 × *g* for 10 min and the supernatant was separated. The total protein concentration in each group was determined by the BCA method, and the optical densities (450 nm) of equal amounts of proteins (50 μL) were determined with a microplate reader and the concentrations were determined by reference to a standard curve. The concentration of Ac-CoA is expressed as ng/mL of protein.

### Western blot analysis

Colonic samples (20 µg proteins) were separated on an 8%, 10%, or 12% sodium dodecyl sulfate polyacrylamide gel and transferred to a polyvinylidene difluoride (PVDF) membrane, which was blocked with 5% non-fat milk and 0.1% Tween-20 in Tris-buffered saline for 1 h and then incubated overnight at 4 °C with primary antibodies against G protein-coupled receptor (GPR) 41 (1:500; Signalway Antibody LLC, College Park, MD, USA), GPR 43 (1:500; Sigma-Aldrich Corporation), histone deacetylation 1/2 (HDAC1/2; 1:1500; Abcam, Cambridge, MA, USA), occludin (1:2000; Abcam), claudin 1 (1:500; Santa Cruz Biotechnology, Inc.), interferon regulatory factor 4 (IRF4; 1:1000; Cell Signaling Technology, Inc., Danvers, MA, USA), NLR family, pyrin domain containing 3 (NLRP3; 1:1000; Abcam), vascular cell adhesion molecule 1 (VCAM1; 1:500; Santa Cruz Biotechnology, Inc.), retinoid acid receptor-related orphan receptor gamma t (RORγt, 1:500; Santa Cruz Biotechnology, Inc.), transforming growth factor β1 (1:1000, Abcam), signal transducer and activator of transcription 3 (STAT3; 1:1000; Cell Signaling Technology), phospho-STAT3 (p-STAT3, Tyr705; 1:1000; Cell Signaling Technology), NADH dehydrogenase subunit 4 (ND4; 1:1000; Signalway Antibody LLC), cytochrome c oxidase subunit 1 (COX1; 1:1000; Abcam), interleukin (IL)-1β (1:500; Invitrogen Corporation, Carlsbad, CA, USA), IL-6 (1:300; Santa Cruz Biotechnology, Inc.), IL-10 (1:300; Santa Cruz Biotechnology, Inc.), IL-17 (1:300; Santa Cruz Biotechnology, Inc.), IL-23 (1:300; Santa Cruz Biotechnology, Inc.), voltage-dependent anion-selective channel 1 (VDAC-1; 1:1000; Santa Cruz Biotechnology, Inc.), glyceraldehyde 3-phosphate dehydrogenase (GAPDH; 1:5000; Abcam), and β-actin (1:5000, Abcam), followed by incubation with a horseradish peroxidase-conjugated goat anti-rabbit or mouse secondary antibody against immunoglobulin G for 1 h at room temperature. The antibody-protein complexes were detected using an enhanced chemiluminescent substrate solution (EMD Millipore Corporation, Billerica, MA, USA) and quantified based on optical density against GAPDH as a control. For mitochondria, VDAC1 was employed as the loading control. The extent of phosphorylation of each protein was evaluated with respect to the abundance of the native form (p-STAT3/STAT3).

### IP

Briefly, 200 µg of colonic homogenate were incubated with 3 µg of IRF4 antibody at 4 °C overnight. Protein G-Sepharose beads (Sigma-Aldrich Corporation) were prewashed three times in IP buffer (10 mMTris-Cl, pH 7.5, 150 mM sodium chloride, 2 mM EDTA, 0.5% Triton-100) for 15 min and incubated with a protein/antibody mixture under constant rotation at 4 °C for 2 h. The precipitant was centrifuged at 10,000 × *g* for 1 min and washed three times with IP buffer to remove nonspecifically bound proteins. Afterward, the immune-complexed beads were resuspended in sodium dodecyl sulfate–polyacrylamide gel electrophoresis loading buffer, heated at 95 °C for 5 min, and then removed by centrifugation at 10,000 × *g*. The supernatants were collected for immunoblot detection of IRF4 and STAT3 with homogenates and without IP buffer as input controls.

### Cell isolation and flow cytometry

Single-lymphocyte suspensions were harvested from the lamina propria colon tissues of rats for flow cytometry, which was conducted with the following gating strategy: lymphocytes → single cells → live cells → CD4 + cells. Briefly, after washing with sterile PBS, the tissues were cut into pieces, which were then subjected to enzymatic treatment using 0.125 mg/mL of Liberase™ Thermolysin Medium (Sigma-Aldrich Corporation) and 0.5 mg/mL of DNase I for 20 min. Cells were filtered through a sieve with 100-μm pores. Filtration of digestive juices was terminated by the addition of 500 μL of fetal bovine serum. The remaining tissue fragments were processed as described above. Cells were collected by filtering through a sieve with 40-μm pores. After centrifugation at 500 × *g* for 5 min at room temperature, the supernatant was discarded and the cells were resuspended in PBS containing 2% fetal bovine serum. Prior to intracellular cytokine staining, 10^6^ cells were stimulated with 50 ng/mL of phorbol 12-myristate 13-acetate and 1 µg/mL of ionomycin (BD Biosciences, San Jose, CA, USA) in the presence of 10 µg/mL of brefeldin A (BD Biosciences) under an atmosphere of 5% CO2/95% air at 37 °C for 5 h. Afterward, the cells were washed with PBS and stained with Fixable Viability Dye eFluor™ 506 (eBioscience, Inc., San Diego, CA, USA) and surface markers at 4 °C in the dark for 30 min, then fixed and permeabilized with eBioscience™ Intracellular Fixation & Permeabilization Buffer for 20 min at room temperature in the dark. For the T-helper 17 (Th17) cell assay, cells were incubated with antibodies against CD4 (eBioscience, clone: OX-35) and IL-17A (eBioscience, clone: eBio17B7). For the regulatory T cell (Treg) assay, cells were incubated with antibodies against CD4, CD25 (eBioscience, clone: OX-39), and FOXP3 (eBioscience, clone: FJK-16 s). For IL-10 detection, cells were incubated with antibodies against CD4 and IL-10 (BD Pharminigen, San Diego, CA, USA, clone: A5-4) or FOXP3 and IL-10, respectively. Finally, data were obtained with a BD FACSCalibur Flow Cytometer (BD Biosciences) and analyzed using FlowJo 7.6 software.

### Electron microscopy

For observation of mitochondria, fresh slices of colon tissue, approximately 1 mm thick, were fixed in 2.5% glutaraldehyde overnight at 4 °C, then washed three times with 0.1 M PBS and postfixed in 1% osmium tetroxide at 4 °C for 2 h. Afterward, the blocks were dehydrated in graded ethanol and embedded in epoxy resin. Randomly selected ultrathin sections were poststained with uranyl acetate and lead citrate and examined under an electron microscope (Koninklijke Philips N.V., Amsterdam, Netherlands).

### Statistical analysis

The microbiome population, targeted metabolomics, and RNA sequencing statistics are described in detail above. Excluding these, the data are presented as the mean ± standard deviation (SD). Statistically significant differences between the means of two or more independent (unrelated) groups were identified by one-way analysis of variance, followed by post-hoc analysis with Dunnett’s test. A probability (*p*) value of less than 0.05 was considered statistically significant (Fig. 1).

## Results

### FMT and replenishment of SCFAs ameliorated CCH-induced changes to the gut microbiota

**Fig. 2:**
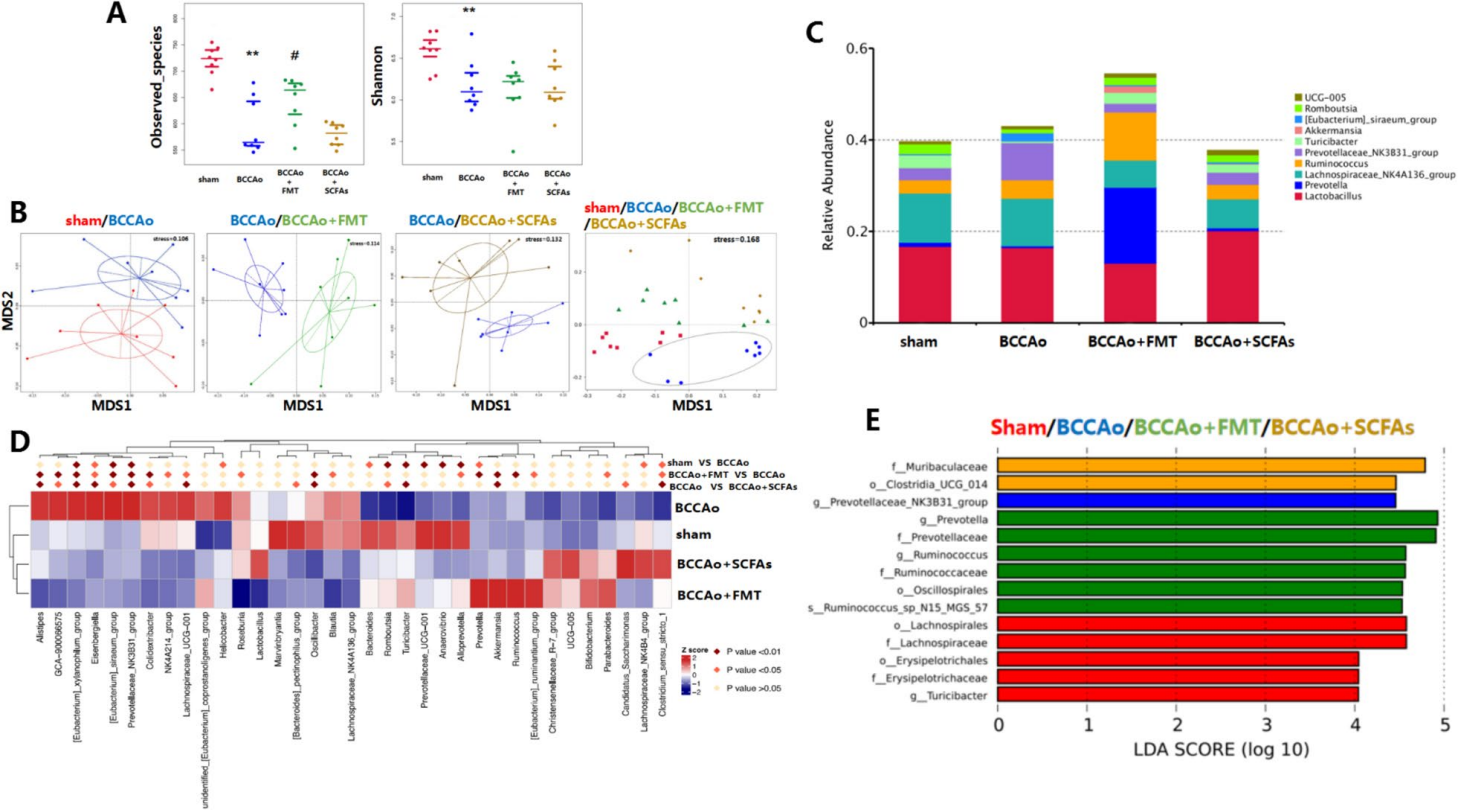
FMT and SCFAs treatment ameliorate CCH-induced gut microbial dysbiosis. **A** Analysis of alpha diversity by shannon index and observed_species. **B** Analysis of beta diversity by non-metric multi-dimensional scaling (NMDS). **C** Relative abundance of top 10 gut microbiota at the genus level. **D** Heatmap based on nonparametric Wilcoxon test by MetaStat showing relative abundance of gut microbiota at the genus level. **E** Analysis of significant difference in abundance of gut bacteria by Linear discriminant analysis (LDA) coupled with effect size (LEfSe). Data are expressed as mean ± SD (*n* = 8 per group). *P < 0.05 vs. sham group. ^#^*P* < 0.05 vs. BCCAo group

Sequencing of the 16S rRNA gene was conducted at 12 weeks after BCCAo. In this study, the observed_species and Shannon diversity indices were used to evaluate the diversity of the gut microbiota. CCH significantly decreased the number of observed species and Shannon diversity (observed species: *p* < 0.001; Shannon: *p* = 0.004). However, FMT partially reversed the decreased number of observed species (*p* = 0.02, Fig. 2A). NMDS analysis was conducted to visualize differences in the taxa of the gut microbiota among the four groups. The results revealed that BCCAo induced a marked difference in gut microbiota composition as compared with the healthy control group, whereas CCH induced significant changes in the gut microbiota of the BCCAo + FMT and BCCAo + SCFAs groups (Fig. 2B). Analysis of similarity using the Bray–Curtis dissimilarity matrix revealed clear compositional differences in gut microbiota between the sham-operated and BCCAo groups, BCCAo and BCCAo + FMT groups, and BCCAo and BCCAo + SCFAs groups. Of the top 10 gut microbiota at the genus level, as compared with the sham-operated group, BCCAo significantly increased the relative abundance of *Prevotellaceae_NK3B31_group* and *[Eubacterium]_siraeum_group*, and lowered that of *Romboutsia*, *Prevotella*, and *Turicibacter*. The relative abundance of *Prevotellaceae_NK3B31_group* was significantly reduced by FMT and replenishment of SCFAs*,* while that of *[Eubacterium]_siraeum_group*, *Prevotella*, and *Turicibacter* was markedly increased. Besides, FMT or administration of SCFAs strikingly increased the CCH-induced relative abundance of *Akkermansia* and *Ruminococcus* (Fig. 2C, D). LEfSe analysis demonstrated that the microbial signatures of the sham-operated, BCCAo, BCCAo + FMT, and BCCAo + SCFAs groups were *Lachnospirales* (order)*/Lachnospiraceae* (family)/ *Erysipelotrichales* (order)/*Erysipelotrichaceae* (family)/*Turicibacter* (genus), *Prevotellaceae_NK3B31_group* (genus), *Prevotellaceae* (family)/*Prevotella* (genus)/*Ruminococcaceae* (family)*/Ruminococcus* (genus)/*Ruminococcus_sp_N15_MGS_57* (species)/*Oscillospirales* (order) and *Clostridia_UCG_014* (order)/*Muribaculaceae* (family), respectively (Fig. 2E).

According to these data, CCH rats developed gut microbial dysbiosis with prolonged CCI by decreasing the richness, diversity, and composition of the gut microbiota, while FMT and SCFA replenishment modulated CCH-induced gut microbial disruption by increasing the abundance of some species, including *Ruminococcaceae* and *Clostridia_UCG_014*.

### FMT and SCFA treatment increased CCH-induced metabolism of SCFAs via regulation of the gut microbiota

**Fig.3:**
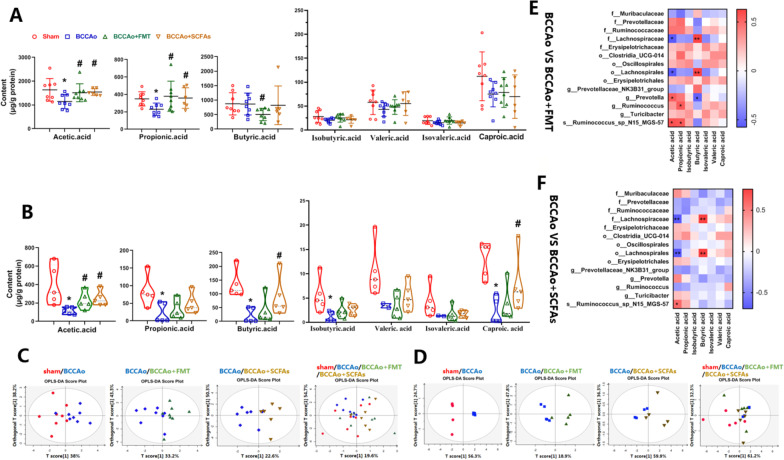
FMT and SCFAs treatment increase CCH-induced metabolite SCFAs. **A** Analysis of fecal SCFAs concentration by gas chromatography-mass spectrometry (GC-MS) (*n* = 8 in sham, BCCAo and BCCAo + FMT group; *n* = 6 in BCCAo + SCFAs group). **B** Analysis of colonic SCFAs concentration by GC-MS (*n* = 5 per group). **C**, **D** Segregation trends of fecal and colonic SCFAs evaluated by orthogonal partial least squares discriminant analysis (OPLS-DA) respectively. **E**, **F** Spearman correlation analysis between gut microbiota and metabolite SCFAs using fecal samples. Data are expressed as mean ± SD. *P < 0.05 vs. sham group. ^#^*P* < 0.05 vs. BCCAo group. In **E**, **F**, *P < 0.05, **P < 0.01

To determine whether changes to the bacterial communities impacted the concentrations of SCFAs in fecal samples and colon tissues, the concentrations of SCFAs were measured with a targeted metabolomics assay. Consistent with the changes to the microbial community structure and composition, CCH, FMT, and SCFAs induced different profiles in SCFAs, especially between the sham-operated and BCCAo, BCCAo and BCCAo + FMT, and BCCAo and BCCAo + SCFAs groups, as determined by OPLS-DA (Fig. 3C, D). Specifically, the concentrations of acetic acid and propionic acid in fecal samples were significantly higher in the BCCAo + FMT and BCCAo + SCFAs groups than the BCCAo group (*p*_FMT_ = 0.046 and 0.036, *p*_SCFAs_ = 0.028 and 0.02, respectively) (Fig. 3A) A similar trend with acetic acid was observed with the colon tissues (*p*_FMT_ = 0.032, *p*_SCFAs_ = 0.008) (Fig. 3B).

To clarify whether the increased metabolism of SCFAs was caused by alterations to the gut microbiota following FMT and SCFA administration, Spearman correlation analysis was conducted using data from the fecal samples. The results showed that acetic acid was positively associated with *Ruminococcus_sp_N15_MGS_57* and *Prevotella*, and negatively related to *Lachnospiraceae* and *Lachnospirales*. Moreover, propionic acid was positively correlated with *Ruminococcus* and *Ruminococcus_sp_N15_MGS_57.* However, interestingly, *Lachnospiraceae* and *Lachnospirales* were positively correlated with butyric acid production, while *Prevotella* was negatively correlated (Fig. 3E, F).

Taken together, these results revealed that the levels of acetic acid and propionic acid were significantly elevated in fecal samples after FMT and SCFA administration. Notably, acetic acid was the only markedly increased metabolite identified in the colon tissues after FMT and SCFA treatment, suggesting that this SCFA might drive the effects of FMT and SCFA treatment against CCH-induced colon dysfunction. *Ruminococcus_sp_N15_MGS_57,* an established gut microbial biomarker after FMT, was positively associated with acetic acid after FMT and SCFA treatment, indicating that *Ruminococcus_sp_N15_MGS_57* may be partially responsible for the increased production of acetic acid after FMT and SCFA treatment in response to CCI.

### FMT and SCFA treatment alleviated CCH-induced impaired colonic barrier

**Fig. 4:**
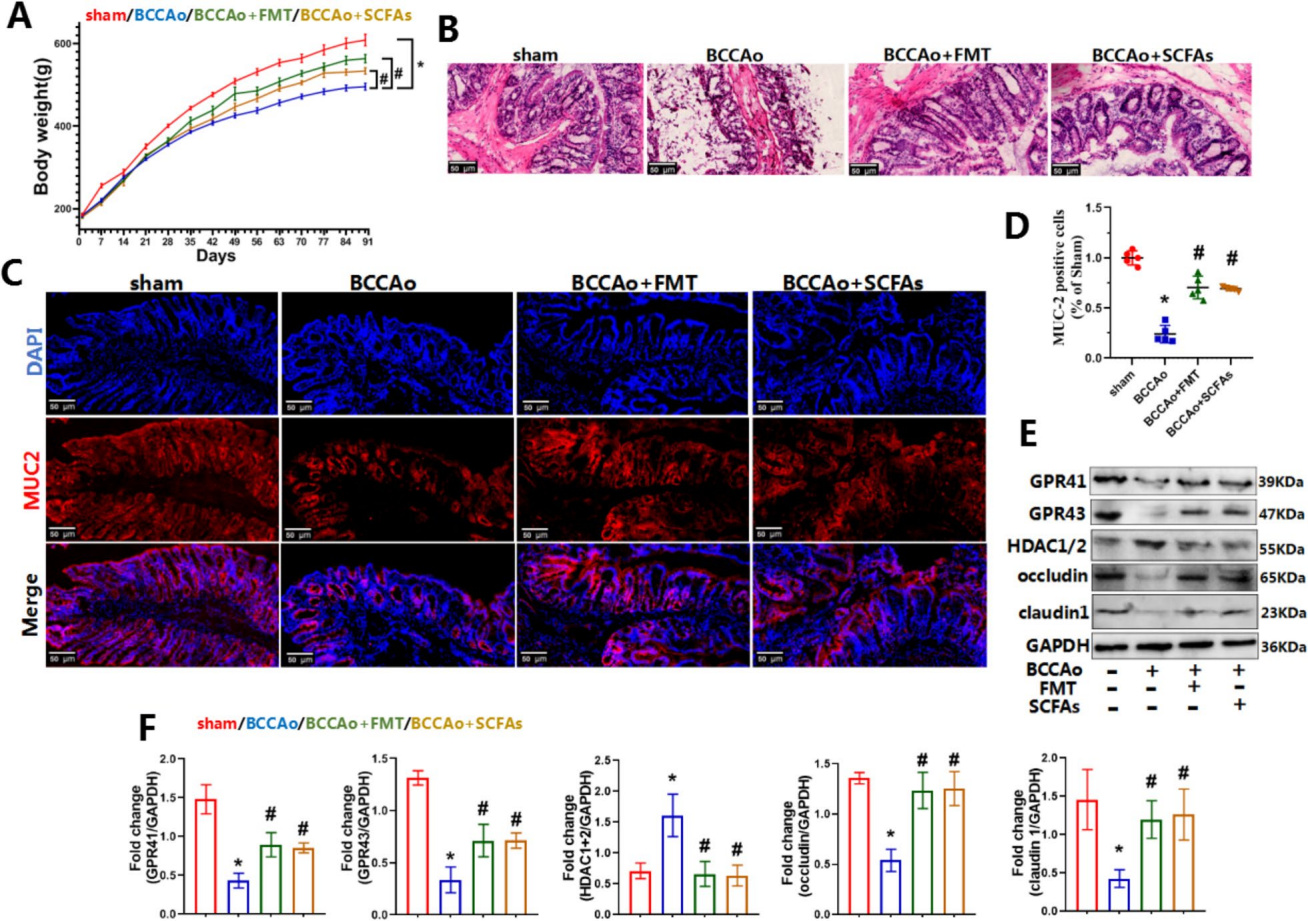
FMT and SCFAs treatment alleviate CCH-induced impaired colonic barrier. **A** Evaluation of body weight. **B** Representative HE staining in colon (scale bars = 50 µm). **C** Representative immunofluorescence staining for MUC2 in colon (scale bars = 50 µm). red: MUC2; blue: DAPI. **D** Relative level of MUC2-positive cells (ratio of sham group, *n* = 5 per group). The average area of MUC2-positive puncta in sham group is set to 1. **E** Representative western blot for GPR41, GPR43, HDAC1/2, occludin, claudin1, and GAPDH in colon. **F** Relative optical density analysis for GPR41, GPR43, HDAC1/2, occludin, claudin1, and GAPDH in colon (*n* = 4 per group). Data are expressed as mean ± SD. *P < 0.05 vs. sham group. ^#^*P* < 0.05 vs. BCCAo group

To determine the effects of FMT and SCFA treatment on colonic barrier function, morphological changes and the expression levels of tight junction proteins and MUC2, as the major component of the colonic mucus layer, were assessed. Furthermore, SCFAs may influence microbe-gut-brain interactions by signaling to the host via G protein-coupled receptors and inhibition of HDAC. Therefore, the protein levels of GPR41, GPR43, and HDAC1/2 were measured in colon tissues. The average weight of male rats at 18 weeks notably decreased from 609 ± 14 to 496 ± 8 g (*p* < 0.01) after BCCAo and then returned to 564 ± 10 g (*p* < 0.01) following FMT treatment and 534 ± 8 g (*p* < 0.01) after SCFA administration (Fig. 4A). Colon tissues stained with HE showed excessive mucosal damage in BCCAo group, which were seldom found in BCCAo + FMT and BCCAo + SCFAs groups (Fig. 4B). Moreover, CCH significantly decreased the number of MUC2-positive cells and the expression levels of tight junction proteins, such as claudin 1 and occludin. Nevertheless, FMT and SCFA replenishment eliminated these changes (Fig. 4C–F). These results indicate that CCH impaired colonic barrier function, markedly reduced the protein levels of GPR41 and GPR43, and aggravated overexpression of HDAC1/2, which were ameliorated by FMT and SCFA treatment (Fig. 4E, F), suggesting alleviation of colonic barrier dysfunction via activation of GPR41 and GPR43 and inhibition of HDAC.

### Inflammation and energy metabolism pathways were not involved in FMT and SCFA treatment after CCH

**Fig. 5:**
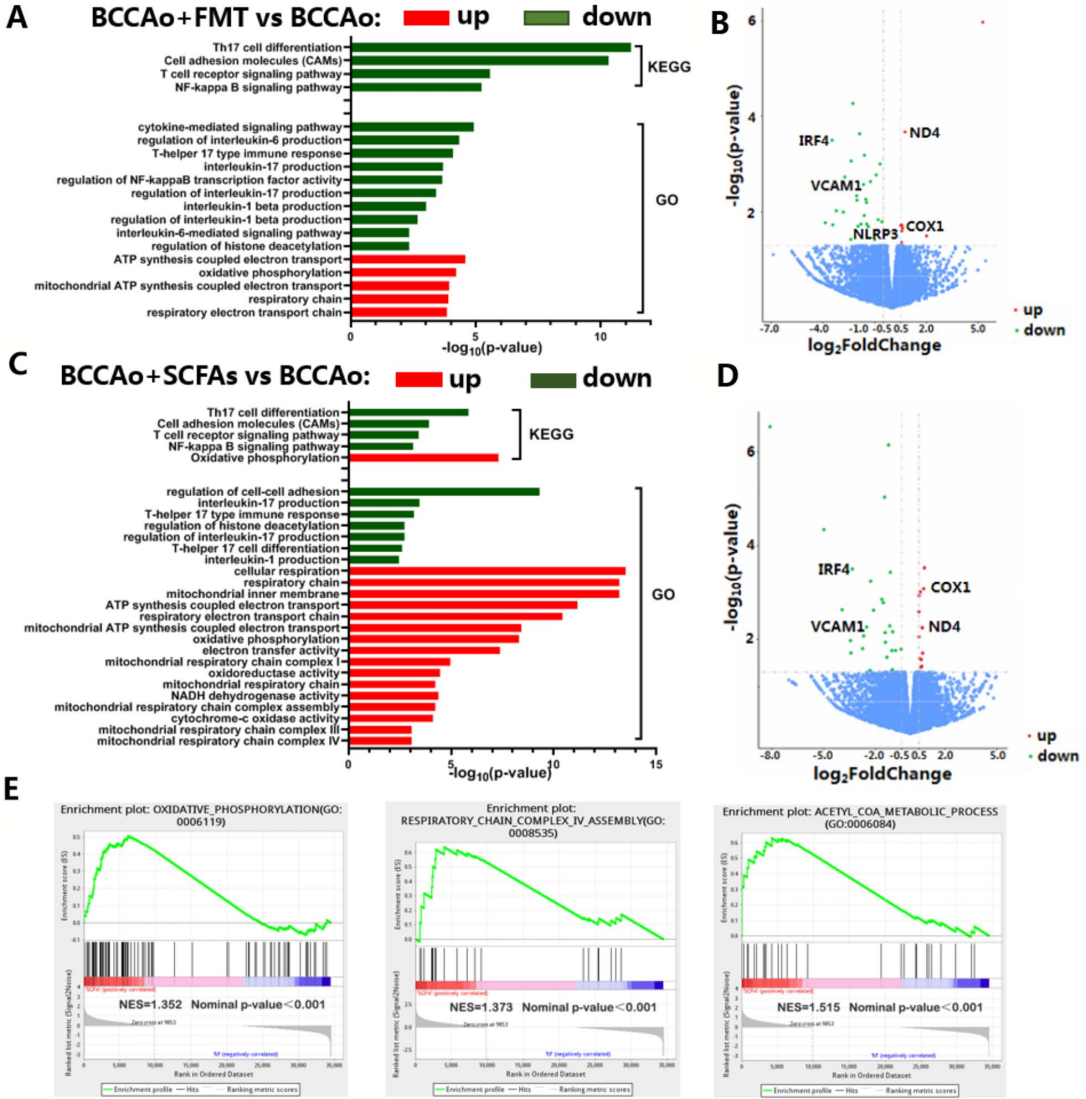
The potential molecular mechanisms involved in the FMT and SCFAs treatment in colon. **A**, **C** Histogram of the significantly enriched GO or KEGG terms in BCCAo group vs. BCCAo + FMT group and BCCAo group vs. BCCAo + SCFAs group respectively according to RNA sequencing datasets. The horizontal coordinate represents − log_10_ (*p*-value), the vertical coordinate represents GO or KEGG names. **B**, **D** Volcano plots showing partial differentially expressed genes (DEGs) in the above pathways we are interested in in BCCAo group vs. BCCAo + FMT group and BCCAo group vs. BCCAo + SCFAs group respectively according to RNA sequencing datasets. The horizontal axis indicates log_2_ (fold change), the vertical axis indicates −log_10_ (*p*-value). Some DEGs passed the verification of western blot are marked with names. **E** Gene set enrichment analysis (GSEA) performed in BCCAo group vs. BCCAo + SCFAs group based on RNA sequencing datasets. The total height of the curve reflects the extent of enrichment, the normalized enrichment score (NES) and norminal *p* value are indicated

Transcriptome analysis was performed to explore the potential molecular mechanisms by which FMT and SCFA treatment promote functional recovery of colonic tissues after CCH. Compared with BCCAo group, GO analysis between the BCCAo + FMT and BCCAo groups showed that FMT was associated with a down-regulation of the pathways related with the production of the cytokines IL-1β, IL-6, and IL-17, the immune response of Th17 cells, and regulation of HDAC, while ATP synthesis coupled electron transport, mitochondrial ATP synthesis coupled electron transport, respiratory ETC, and oxidative phosphorylation were markedly enhanced (Fig. 5A). Furthermore, KEGG analysis illustrated that FMT strikingly inhibited Th17 cell differentiation, cell adhesion molecules, and the T cell receptor and NF-kappa B (NF-κB) pathways (Fig. 5A). Similar to FMT, SCFA replenishment significantly weakened regulation of cell–cell adhesion, IL-1 and IL-17 production, differentiation and immune response of Th17 cells, and regulation of HDAC pathways, but notably strengthened ATP synthesis coupled electron transport, mitochondrial ATP synthesis coupled electron transport, respiratory electron transport chain, mitochondrial respiratory chain complex I/III/IV, oxidative phosphorylation, oxidoreductase activity, NADH dehydrogenase activity, and cytochrome-c oxidase activity (Fig. 5C). Moreover, SCFA treatment significantly attenuated Th17 cell differentiation, cell adhesion molecules, and the T cell receptor and NF-κB pathways, but promoted oxidative phosphorylation (Fig. 5C). In addition, GSEA found that SCFA treatment significantly modified pathways associated with the Ac-CoA metabolic process, oxidative phosphorylation, and respiratory chain complex IV (Fig. 5E). These results indicate that both SCFA treatment and FMT were associated with signaling of IL-1β, IL-6, and IL-17, Th17 cell differentiation, regulation of HDAC, cell adhesion molecules, NF-κB pathway, ATP synthesis coupled electron transport, respiratory ETC, and oxidative phosphorylation.

Overall, 850 differentially expressed genes (DEGs) were upregulated and 330 were downregulated between the BCCAo + FMT and BCCAo groups, while 876 DEGs were upregulated and 1395 were downregulated between the BCCAo + SCFAs and BCCAo groups. Of note, the DEGs ND4, COX1, IRF4, NLRP3, and VCAM1 verified by western blot analysis were associated with cytokine-mediated signaling, including production of IL-1β and IL-17, Th17 cell differentiation, cell adhesion molecules, respiratory ETC, and oxidative phosphorylation (Figs. 8A and 9F).

### FMT and SCFA treatment regulated the balance between Tregs and Th17 cells

**Fig. 6:**
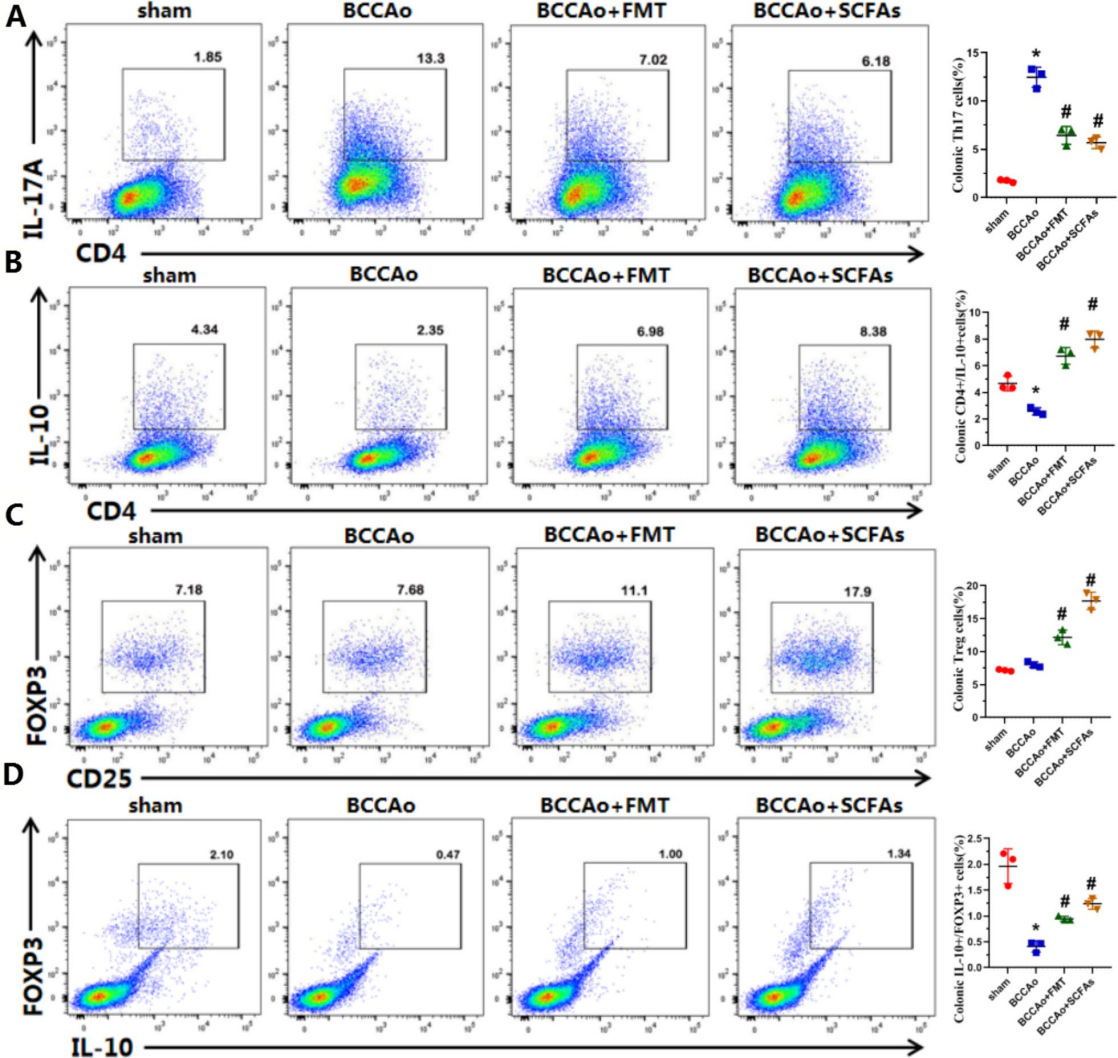
FMT and SCFAs treatment improve colonic Treg/Th17 balance. **A** Representative plot of Th17 cells analyzed by flow cytometry and scatter diagram of the percentage of Th17 cells in colon. **B** Representative plot of CD4^+^IL-10^+^ cells analyzed by flow cytometry and scatter diagram of the percentage of CD4^+^IL-10^+^ cells in colon. **C** Representative plot of Treg cells analyzed by flow cytometry and scatter diagram of the percentage of Treg cells in colon. **D** Representative plot of FOXP3^+^IL-10^+^ cells analyzed by flow cytometry and scatter diagram of the percentage of FOXP3^+^IL-10^+^ cells in colon. Data are expressed as mean ± SD (*n* = 3 per group). *P < 0.05 vs. sham group. ^#^*P* < 0.05 vs. BCCAo group

The significant modification of cytokine-mediated signaling, including IL-1β, IL-6, and IL-17 production and Th17 cell differentiation pathways after FMT and SCFA treatment demonstrated that Treg/Th17 balance plays a fundamental role in stabilizing immune homeostasis in colon tissues. To reveal the impact of FMT and SCFA treatment on the balance of Tregs and Th17 cells, colon tissues were harvested from all four groups for flow cytometry. The relative abundance of each cell type was reported as a percent of CD4^+^ T cells. The results showed that CCH markedly elevated the percentage of Th17 cells, which was remarkably decreased by FMT and SCFA treatment (Fig. 6A), but had no obvious effect on the percentage of Tregs, while FMT and SCFA treatment remarkably increased the percentage of Tregs (Fig. 6C). IL-10 is the key cytokine in Treg-mediated inflammatory suppression, thus the proportions of CD4^+^IL-10^+^ and FOXP3^+^IL-10^+^ cells were subsequently evaluated. CCH reduced the proportions of CD4^+^IL-10^+^ and FOXP3^+^IL-10^+^ cells, which was reversed by FMT and SCFA treatment (Fig. 6B, D), demonstrating that FMT and SCFA treatment promoted IL-10 expression in effector T cells, especially Tregs, after CCH. In summary, FMT and SCFA replenishment upregulated the proportion of colonic Tregs and downregulated the ratio of colonic Th17 cells in response to CCH-induced inflammation of colonic tissues, thereby improving the balance of Tregs and Th17 cells.

**Fig. 7:**
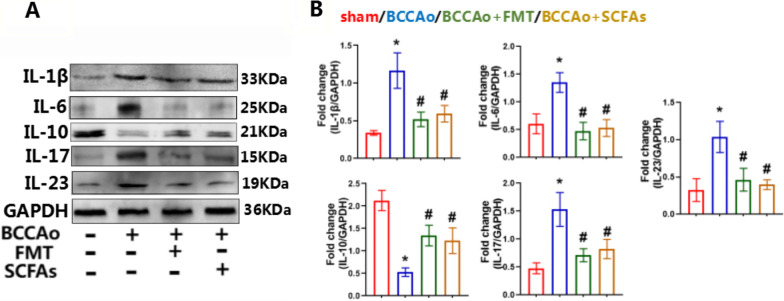
FMT and SCFAs treatment decrease pathogenic- Th17 cells differentiation. **A** Representative western blot for IL-1β, IL-6, IL-10, IL17A, IL-23 and GAPDH in colon. **B** Relative optical density analysis for IL-1β, IL-6, IL-10, IL17A, IL-23 and GAPDH in colon. Data are expressed as mean ± SD (*n* = 4 per group). *P < 0.05 vs. sham group. ^#^*P* < 0.05 vs. BCCAo group

**Fig. 8:**
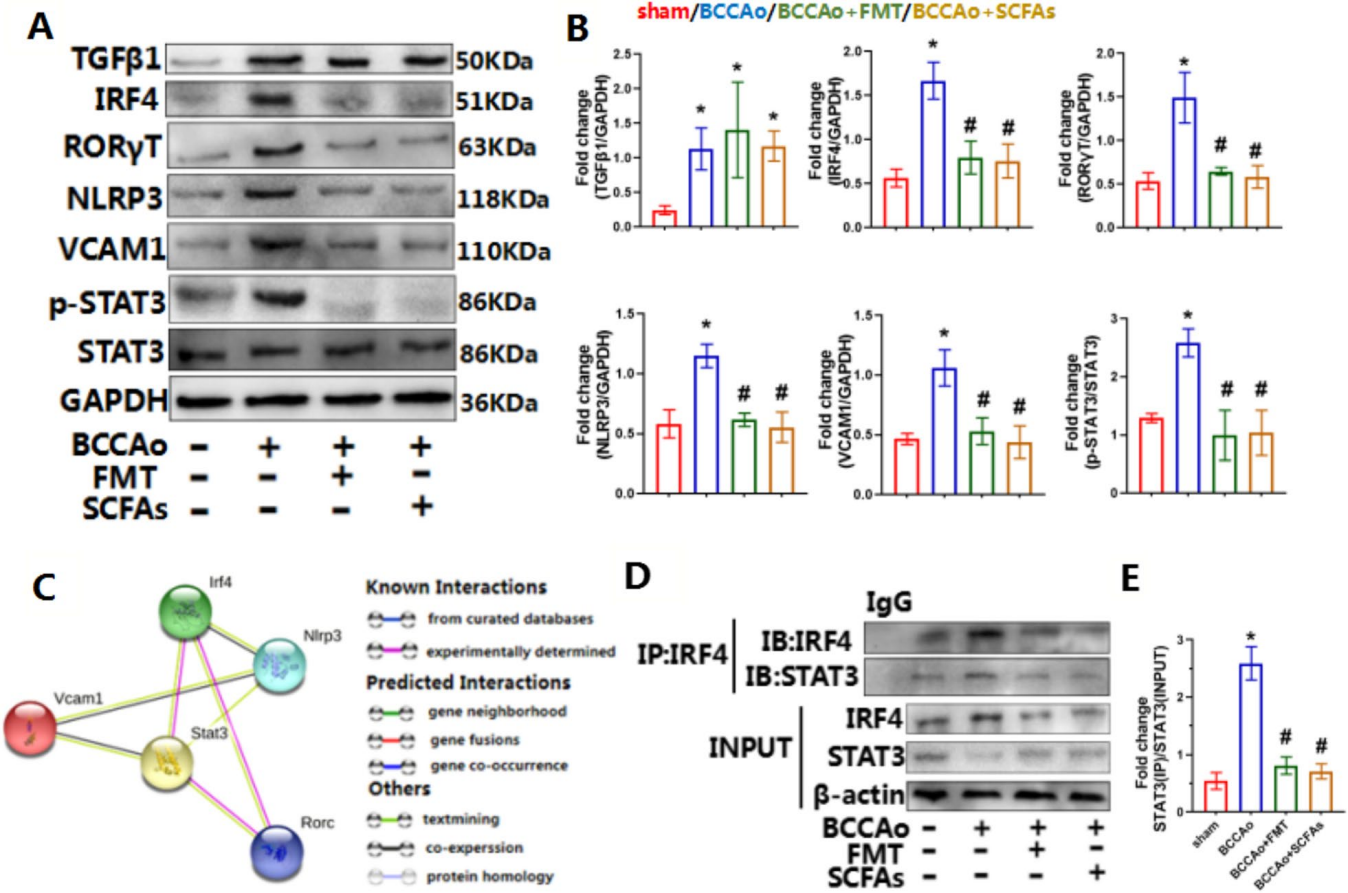
FMT and SCFAs treatment inhibit CCH-induced inflammatory IL-17 pathways in colon. **A** Representative western blot for TGFβ1, IRF4, RORγT, NLRP3, VCAM1, p-STAT3, STAT3 and GAPDH in colon. **B** Relative optical density analysis for TGFβ1, IRF4, RORγT, NLRP3, VCAM1, p-STAT3, STAT3 and GAPDH in colon (*n* = 4 per group). **C** The protein–protein interaction network downloaded from the STRING database indicated the interactions among the candidate genes. **D** Representative immunoprecipitation (IP) for IRF4, STAT3 and GAPDH in colon. (E) Relative ratio of STAT3 in IP complex and STAT3 in whole cell lysates in colon (*n* = 3 per group). The relative abundance of STAT3 in IP complex is determined by western blotting, and compared to the level of STAT3 in whole cell lysates. Data are expressed as mean ± SD. *P < 0.05 vs. sham group. ^#^*P* < 0.05 vs. BCCAo group

Recent evidence indicates that cytokines regulate the differentiation of pathogenic and non-pathogenic Th17 cells [28]. IL-1β + IL-6 + IL-23 pathogenic Th17 cells produce high amounts of cellular adhesion molecules, including VCAM1, and low amounts of immune-regulatory molecules, including IL-10 [29, 30]. To further clarify the effects of FMT and SCFA treatment on differentiation of pathogenic Th17 cells in vivo in response to CCH, western blot analysis was conducted to measure the protein levels of IL-1β, IL-6, IL-10, IL-17A, IL-23, and VCAM1 in colon tissues. The results showed that CCH strikingly increased protein levels of IL-1β, IL-6, IL-17A, IL-23, and VCAM1 and decreased protein expression of IL-10 (Figs. 7 and 8A, B), suggesting that CCH exacerbated expression of pathogenic Th17 cells and promoted inflammation of colon tissues, which were revered by FMT and SCFA administration, demonstrating that FMT and SCFA treatment weakened differentiation of pathogenic-Th17 cells and mitigated inflammation of colon tissues.

### FMT and SCFA treatment suppressed inflammatory IL-17 signaling

Transcription factor IRF4 is essential for differentiation of Th17 cells [31]. Upon Th17 cell-skewing stimulation, IRF4 induces chromatin remodeling, followed by RORγt-mediated transcription, which are essential for initiating production of IL17 [32]. Hence, the protein levels of IRF4 and RORγt in colon tissue were measured. Consistent with IRF4 mRNA levels, as determined by RNA-seq analysis, CCH significantly increased protein expression of IRF4, which was markedly attenuated by FMT and SCFA treatment (Fig. 8A, B), indicating that transcription factor IRF4 might be an important modulator involved in FMT and SCFA treatment of CCH-induced chronic inflammation of colon tissues. Interestingly, the protein level of RORγt showed the same tendency as IRF4 even though there was no significant change, as determined by RNA-seq analysis. CCH substantially promoted the production of IL-1β^+^IL-6^+^IL-23^+^ pathogenic Th17 cells. The protein levels of NLRP3, which reportedly promotes the generation of IL-1β [33], were further measured in colon tissues. In accordance with the results of RNA-seq analysis, CCH dramatically elevated the protein levels of NLRP3, confirming the generation and activation of IL-1β. However, FMT and SCFA administration markedly downregulated NLRP3 (Fig. 8A, B), indicating that this treatment strategy may partly decrease differentiation of pathogenic Th17 cells by reducing the production of IL-1β. Moreover, all chronic inflammatory disorders are characterized by rapid recruitment and often inappropriate retention of inflammatory cells facilitated by the interactions of chemokine receptors with their respective endothelial and mucosal ligands, which consist of members of the immunoglobulin superfamily of cellular adhesion molecules [34, 35]. Hence, protein expression of the cellular adhesion molecule VCAM1, which was identified as a significant DEG by RNA-seq analysis, was subsequently assessed in colon tissues. FMT and SCFA administration notably reversed CCH-induced upregulation of VCAM1 protein expression (Fig. 8A, B), further confirming the inhibitory effects of FMT and SCFA replenishment on CCH-induced chronic inflammation of colon tissues.

### FMT and SCFA treatment prevented CCH-induced interactions of the IRF4/STAT3 complex

To explore the potential interactions among candidate proteins, functional enrichment analysis of protein–protein interaction networks was conducted using the STRING database. The results demonstrated that the transcription factor STAT3 might play an important role in these interactions (Fig. 8C). Co-IP and MS analyses were performed to further determine whether these molecular mechanisms of FMT and SCFA treatment were involved in the differentiation of pathogenic Th17 cells and inflammatory signaling of IL-17, which showed that STAT3 interacted with IRF4 following CCH. This result was confirmed by the IP with an antibody against IRF4 (Fig. 8D, E). The relative abundance of STAT3 in the IRF4/STAT3 complex was compared to that in whole-cell lysate. As compared with the sham-operated group, CCH significantly increased the level of STAT3 in IP complex/STAT3 in whole-cell lysate. Notably, there was no significant difference in the total abundance of STAT3 between the sham-operated and CCH groups, while the level of p-STAT3 was markedly increased after CCH treatment. These results showed that CCH promoted formation of the IRF4/STAT3 complex and phosphorylation of STAT3 (Fig. 8A, B, D, E), leading to chronic inflammation of colon tissues. However, FMT and SCFA treatment induced the dissociation of IRF4 and STAT3 and decreased the phosphorylation of STAT3, resulting in inhibition of chronic inflammation of colon tissues.

### FMT and SCFA treatment mitigated mitochondrial dysfunction

**Fig. 9:**
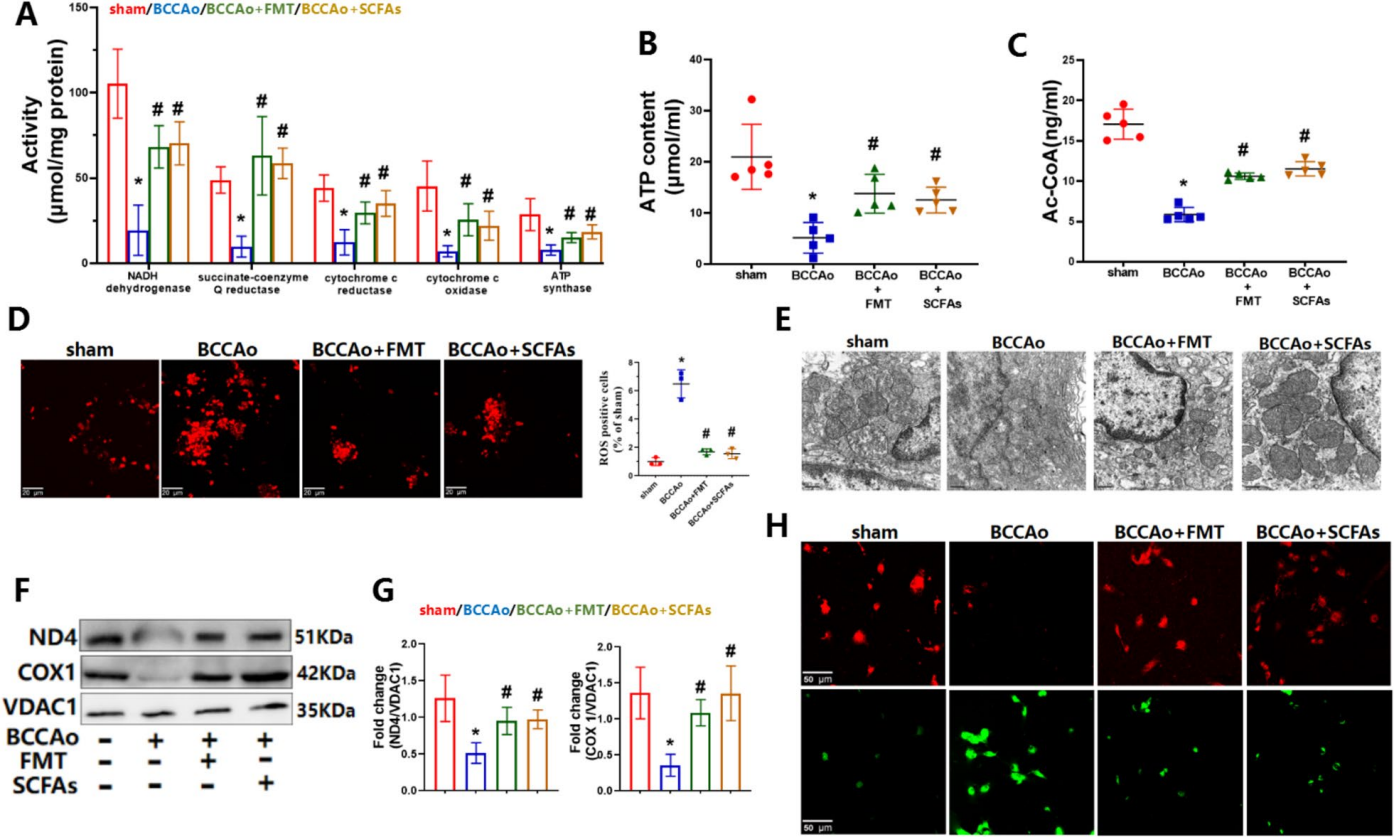
FMT and SCFAs treatment relieve CCH-induced colonic mitochondrial dysfunction. **A** The activities of electron transport chain complex I-V in colon (*n* = 5 per group). **B** The content of ATP in colon (*n* = 5 per group). **C** The content of acetyl coenzyme A in colon (*n* = 5 per group). **D** Representative DHE fluorescence staining for ROS and relative level of ROS in colon (ratio of sham group, n = 3 per group, scale bars = 20 μm). The average area of ROS-positive puncta in sham group is set to 1. **E** Representative electron micrographs of mitochondria in colon (scale bars = 0.5 µm). **F** Representative western blot for ND4,COX1 and VDAC1 in colon. **G** Relative optical density analysis for ND4,COX1 and VDAC1 in colon (n = 4 per group). **H** Representative JC-1 fluorescence staining for mitochondrial membrane potential (scale bars = 50 µm). Impaired mitochondria: low intensity of red fluorescence but high intensity of green fluorescence. Healthy mitochondria: high intensity of red fluorescence but low intensity of green fluorescence. Data are expressed as mean ± SD. *P < 0.05 vs. sham group. ^#^*P* < 0.05 vs. BCCAo group

Considering that modules upregulated in the RNA-seq dataset were mainly enriched in mitochondrial respiratory ETC and oxidative phosphorylation after FMT and SCFA treatment, mitochondrial membrane potential, ultrastructural changes, and ROS production were evaluated in colon tissues. As compared with the sham-operated group, CCH significantly increased ROS accumulation, but decreased the ratio of red/green fluorescence intensity induced by staining for JC-1 (Fig. 9D, H). Analysis of the mitochondrial ultrastructure showed that CCH induced abnormal mitochondrial swelling and vague cristae (Fig. 9E), thereby confirming mitochondrial dysfunction. Then, to determine whether and how FMT and SCFA treatment regulate mitochondrial ETC function, the activities of ETC complexes I–V and ATP content were assessed in colon tissues. As compared to the sham-operated group, CCH administration markedly reduced the activities of NADH dehydrogenase (complex I), succinate-coenzyme Q reductase, cytochrome c reductase, cytochrome c oxidase, and ATPase and the subsequent decrease in ATP content (Fig. 9A, B). Acetate was the prominent metabolite of SCFAs following FMT and SCFA treatment in this study. As administration of CCH can result in a state of hypoxia or glucose deprivation, acetate could serve as an important source of Ac-CoA [36], which participates in numerous biochemical reactions. GSEA found that Ac-CoA metabolism was significantly modified after SCFA treatment (Fig. 5E). Thus, the Ac-CoA content was detected in colon tissues. The results showed that CCH strikingly decreased Ac-CoA levels, which were normalized by FMT and SCFA treatment (Fig. 9C). Collectively, these results indicate that CCH impaired mitochondrial ETC and oxidative phosphorylation. Subsequently, protein levels of the significantly enriched DEGs ND4 and COX1 (complex subunits I and IV, respectively) were measured in colon tissues (Fig. 9F, G). The results showed that CCH remarkably decreased protein expression of ND4 and COX1 as compared to the sham-operated group, confirming that ND4 and COX1 are critical to CCH-induced damage to mitochondrial ETC and oxidative phosphorylation. Nevertheless, CCH-induced mitochondrial injury was strongly alleviated by FMT and SCFA administration, suggesting protective effects by mitochondrial metabolic reprogramming in response to chronic ischemia.

## Discussion

To the best of our knowledge, the present study is the first to report that FMT and SCFA replenishment ameliorated CCH-induced gut microbial dysbiosis by increasing the proportion of Ruminococcus_sp_N15_MGS_57, which may promote microbial metabolism of SCFAs, resulting in increased production of acetic acid and subsequent alterations to mitochondrial energy metabolism in colon tissues by promoting mitochondrial ETC and oxidative phosphorylation. Furthermore, FMT and SCFA administration reversed the CCH-induced imbalance in Tregs and Th17 cells by inhibiting differentiation of pathogenic Th17 cells and the IL-17 pathway, resulting in alleviation of impaired barrier function and chronic inflammation in colon tissues. In aggregate, these results indicate that FMT and SCFA replenishment constitute a promising therapeutic strategy for the treatment of colonic dysfunction due to CCI.

Prior reports revealed the impact of acute ischemic stroke on gut dysfunction and intestinal dysbiosis, highlighting the delicate interplay between intestinal injury and the intestinal microbiota [18, 20, 37, 38]. However, at present, there is little evidence supporting the effects of CCI on intestinal damage and gut microbial dysbiosis. A previous study found that cerebral infarction promoted persistent gut microbial dysbiosis, intestinal mucosal damage, and chronic systemic inflammation in cynomolgus monkeys at 6–12 months [15]. Consistently, the results of the present study revealed that CCH induced gut microbial dysbiosis by lowering the abundance of the genera *Romboutsia*, *Turicibacter*, and *Prevotella,* and reducing the concentrations of SCFAs, especially acetic acid and propionic acid, accompanied by impaired colonic barrier function and chronic colonic inflammation. Nevertheless, FMT and oral SCFA administration induced a shift in the composition of the gut microbiota and concentrations of SCFAs by elevating the relative abundances of *Akkermansia*, *Turicibacter*, *Ruminococcus*, and *Prevotella*, and increasing concentrations of acetic acid and propionic acid in feces as well as the concentration of acetic acid in the colon, accompanied with restoration of colonic barrier function and decreased colonic inflammation. Of note, FMT and oral administration of SCFAs significantly upregulated the concentrations of acetic acid and propionic acid in feces, while only the acetic acid content was increased in colon tissues, which might be attributed to dysfunction of the colonic barrier because impairment may block absorption of SCFAs from feces to colon tissues. As the only SCFA remarkably increased in colon tissues, acetic acid might be the main driver of the protective effects of FMT and SCFA administration against CCH-induced colonic dysfunction. Furthermore, the microbial signature was *Prevotellaceae* (family)/*Prevotella* (genus)/*Ruminococcaceae* (family)*/ Ruminococcus* (genus)/*Ruminococcus_sp_N15_MGS_57* (species)/*Oscillospirales* (order) after FMT, indicating that these intestinal microbes might be associated with elevated concentrations of acetic acid. Subsequent Spearman correlation analysis verified a significant positive correlation between the abundance of *Ruminococcus_sp_N15_MGS_57* and production of acetic acid, suggesting that both are likely important factors for treatment of CCH-induced colonic dysfunction.

Tregs act as dedicated inhibitors of diverse inflammatory responses through Th17 suppression and anti-inflammatory cytokine IL-10 secretion [39]. Acute ischemic stroke was reported to promote an imbalance between peripheral Tregs and Th17 cells and enhance IL-17A production to maintain a state of inflammation [40]. Accordingly, in a state of CCI, CCH induced the same tendency in colon tissues. Moreover, IL-1β, IL-6, and IL-23 were found to drive differentiation of pathogenic Th17 cells, while TGF-β1 and IL-6 promoted differentiation of non-pathogenic Th17 cells [29]. In the present study, there were no obvious differences in TGF-β1 mRNA and protein levels between the BCCAo, BCCAo + FMT, and BCCAo + SCFAs groups, whereas expression levels of IL-1β, IL-6, IL-23, and VCAM1 were strikingly increased by CCH administration, but dramatically reduced after FMT and SCFA administration, indicating that this strategy eliminated CCH-induced colonic inflammation by decreasing differentiation of pathogenic Th17 cells. With respect to IL-10 generation, evidence illustrates that IL-10 is critical for Treg-mediated suppression of γδT cells [41]. In the present study, FMT and SCFA treatment reversed CCH-induced alterations to the proportions of CD4^+^IL-10^+^ and FOXP3^+^IL-10^+^ cells, suggesting that IL-10 might play an important role in alleviating colonic inflammation in response to CCH and, thus, presents a potential therapeutic target.

In a state of CCH, the activated NLRP3 inflammasome regulates the production of IL-1β [42]. In the present study, FMT and SCFA treatment substantially reduced the expression levels of NLRP3 and IL-1β, indicating that this intervention could also downregulate the production of IL-1β by inhibiting activation of the NLRP3 inflammasome, thereby leading to decreased differentiation of pathogenic Th17 cells. IL-6-mediated loss of suppressive function of Tregs against inflammation requires phosphorylation of STAT3 [43]. CCH significantly elevated expression of IL-6 and p-STAT3, confirming that phosphorylation of STAT3 may influence the balance between Tregs and Th17 cells after CCI. However, FMT and SCFA administration reversed this phenomenon, indicating the protective effects of Treg function by FMT and SCFA replenishment. IRF4, which is critical to the generation of IL-17-producing Th17 cells [44], acts as a master regulator of Th17 cell differentiation in the intestinal mucosa by increasing production of IL-6, IL-17A, IL-22, and RORγt [45]. Recent data highlights IRF4 as a putative molecular master switch of transcriptional regulators that drives chronic intestinal inflammation through both T cell-intrinsic and -extrinsic mechanisms [46]. In addition, increased IRF4 expression was reported to quench pro-neuroinflammatory responses and improve stroke outcomes [47], suggesting that the function of IRF4 is modified under different conditions. In the present study, IRF4 expression was markedly enhanced in colon tissues in response to CCI. Furthermore, CCH promoted the formation of the IRF4/STAT3 complex and the phosphorylation of STAT3, indicating that the IRF4/STAT3 complex might induce activation of STAT3 in colon tissues. In contrast, FMT and SCFA treatment promoted dissociation of STAT3 from IRF4 and abolished overexpression of IRF4 and phosphorylation of STAT3, indicating the protective effects of FMT and SCFA replenishment against IRF4-mediated chronic colonic inflammation.

In the mitochondria, NADH generated in the tricarboxylic acid cycle enters the ETC, leading to oxidative phosphorylation and subsequent production of ATP, which is important to the physiological and biochemical activities of all cells [48]. CCH is known to exacerbate mitochondrial dysfunction in diseases involving the central nervous system [49] and shown to promote similar effects in colon tissues in the present study. Moreover, CCH inhibited the activities of mitochondrial ETC complexes I–V, thereby reducing ATP generation and eventually resulting in lowered energy production by the mitochondria. Acetate was the prominent metabolite of the SCFAs following FMT and SCFA administration and, thus, could serve as an important source of Ac-CoA in a state of hypoxia or glucose deprivation [36]. Inhibition of Ac-CoA production could lead to inhibition of oxidative phosphorylation and mitochondrial ATP generation [50]. The results of the present study illustrate that CCH-induced reductions in Ac-CoA levels were markedly elevated by FMT and SCFA administration, accompanied by increased oxidative phosphorylation and ATP production, indicating the protective effects of FMT and SCFA replenishment against dysregulation of mitochondrial energy metabolism. Hence, Ac-CoA generated by acetic acid might be partly responsible for the change in CCH-induced oxidative phosphorylation, thereby presenting a potential target to counteract dysregulation of mitochondrial energy metabolism in colon tissues in response to CCI. In addition, the protein expression levels of complex I subunit ND4 and complex IV subunit COX1 were significantly downregulated by CCH and upregulated by FMT and SCFA administration, suggesting that ND4 and COX1 may be critical targets of FMT and SCFA treatment against mitochondrial ETC and impaired oxidative phosphorylation induced by CCH.

Histone acetyltransferase (HAT) and HDAC are enzymes that control the state of histone acetylation. Long-held belief indicates that SCFAs promote histone acetylation by inhibiting HDAC [51], which was also found in our present study. However, a recent study reported that administration of SCFAs induced histone acetylation via activation of HAT rather than inhibition of HDAC, and propionic acid and butyric acid boosted histone acetylation via p300 HAT, while acetic acid had negligible effects on HAT-induced histone acetylation [52]. Although the HAT-mediated effects of acetic acid had no effect on histone acetylation, microbiota-derived acetic acid is reported to promote intestinal innate immunity via the Tip60 HAT complex [53]. Collectively, these results confirm that HAT may play an important role in SCFA-induced histone acetylation and, thus, presents a potential target for regulating the effects of SCFAs. However, future studies are needed to clarify the activities of HAT in a state of CCH.

There were several limitations to this study that should be addressed. First, the effects of FMT and SCFA treatment on brain tissues after CCH remain unclear. Second, cerebral blood flow was not measured in this study, which prevented assessment of successful ischemia among the three BCCAo groups. Third, 16S rRNA gene expression analysis after antibiotic treatment is needed to verify that antibiotic treatment depleted the gut microbiota. Furthermore, considering that antibiotic treatment or salt alone could affect gut dysfunction, control groups would be needed to determine whether antibiotic treatment or salt-matched control had amplified the effects of FMT and SCFA treatment, respectively. Fourth, only colon tissues were examined, thus further studies are needed to validate the effects in colonic Th17 cells. Fifth, although there is presently no appropriate cell model of CCI, determination of the oxygen consumption rate using appropriate CCI cells would effectively complement the results of oxidative phosphorylation. Lastly, SCFA treatment not only inhibited the production of pro-inflammatory cytokines, but also prevented migration and recruitment of immune cells to endothelial cells, which is an important step in the development of inflammatory diseases [54]. Since, colonic epithelial cells might employ different mechanisms than immune cells, future studies are needed to explore the effects of SCFAs on colonic epithelial cells and immune cells.

## Conclusions

In summary, these findings showed that CCH modified the composition of the gut microbiota and the production of metabolites of SCFAs that might be associated with inhibition of mitochondrial ETC activities and oxidative phosphorylation, leading to dysregulation of mitochondrial energy metabolism. Besides, CCH induced differentiation of pathogenic Th17 cells, formation of IRF4/STAT3 complexes, and phosphorylation of STAT3, in association with an impairment of the colonic barrier and chronic colonic inflammation. In contrast, FMT and SCFA replenishment ameliorated CCH-induced gut microbial dysbiosis by increasing the intestinal abundance of *Ruminococcus_sp_N15_MGS_57* and modulating microbial metabolism of SCFAs by increasing the acetic acid content. This was associated by an improvement of the balance between Tregs and Th17 cells, mitochondrial ETC activities, and oxidative phosphorylation to prevent colonic inflammation and dysregulation of mitochondrial energy metabolism in response to CCH. Collectively, these findings highlight the potential therapeutic effects of FMT and SCFA replenishment against colonic dysfunction after CCI.

## Data Availability

The 16S rRNA gene sequencing and RNA-seq data were uploaded in BioProject database (https://www.ncbi.nlm.nih.gov/bioproject) with accession numbers PRJNA869931 and PRJNA781099. Due to the further use by our research group, the other data used in this article are available from the corresponding author upon reasonable request and subject to confidentiality if necessary.

## Abbreviations

CCH: Chronic cerebral hypoperfusion
SCFAs: Short-chain fatty acids
FMT: Fecal microbiota transplantation
BCCAo: Bilateral common carotid artery’s occlusion
HDAC: Histone deacetylation
GC-MS: Gas chromatography-mass spectrometry
DEGs: Differential genes
KEGG: Kyoto encyclopedia of genes and genomes
GO: Gene ontology
GSEA: Gene set enrichment analysis
ATP: Adenosine triphosphate
NADH-DH: NADH dehydrogenase
SQR: Succinate-coenzyme Q reductase
CCR: Cytochrome c reductase
CCO: Cytochrome c oxidase
ATPase: ATP synthase
ETC: Electron transport chain
HE: Hematoxylin and eosin
DHE: Dihydroethidium
ELISA: Enzyme-linked immunosorbent assay
IRF4: Interferon regulatory factor 4
NLRP3: NLR family, pyrin domain containing 3
VCAM1: Vascular cell adhesion molecule 1
TGFβ1: Transforming growth factor β1
COX1: Cytochrome c oxidase subunit 1
ND4: NADH dehydrogenase subunit 4
RORγt: Retinoid acid receptor-related orphan receptor gamma t
STAT3: Signal transducer and activator of transcription 3
FOXP3: Forkhead box P3
